# Brain Region- and Age-Dependent 5-Hydroxymethylcytosine Activity in the Non-Human Primate

**DOI:** 10.3389/fnagi.2022.934224

**Published:** 2022-07-13

**Authors:** Yanru Xu, Liying Zhong, Huixian Wei, Yuwei Li, Jiaxiang Xie, Leijie Xie, Xiusheng Chen, Xiangyu Guo, Peng Yin, Shihua Li, Junwei Zeng, Xiao-Jiang Li, Li Lin

**Affiliations:** ^1^Guangdong Key Laboratory of Nonhuman Primate Models of Human Diseases, Key Laboratory of CNS Regeneration (Ministry of Education), Guangdong-Hongkong-Macau Institute of CNS Regeneration, Jinan University, Guangzhou, China; ^2^Guangdong Provincial People's Hospital, Guangdong Academy of Medical Sciences, Guangzhou, China

**Keywords:** non-human primates, epigenetics, brain region, aging, neurodegenerative diseases

## Abstract

Because of the difficulty in collecting fresh brains of humans at different ages, it remains unknown how epigenetic regulation occurs in the primate brains during aging. In the present study, we examined the genomic distribution of 5hmC, an indicator of DNA methylation, in the brain regions of non-human primates (rhesus monkey) at the ages of 2 (juvenile), 8 (young adult), and 17 (old) years. We found that genomic 5hmC distribution was accumulated in the monkey brain as age increased and displayed unique patterns in the cerebellum and striatum in an age-dependent manner. We also observed a correlation between differentially hydroxymethylated regions (DhMRs) and genes that contribute to brain region-related functions and diseases. Our studies revealed, for the first time, the brain-region and age-dependent 5hmC modifications in the non-human primate and the association of these 5hmC modifications with brain region-specific function and potentially aging-related brain diseases.

## Introduction

Aging is an intrinsic and complex biological process that is characterized by structural degeneration and functional decline, progressively leading to aging-related diseases, such as neurodegenerative diseases in humans, including Parkinson's disease, trinucleotide repeat disorders, and Alzheimer's disease (Morrison and Hof, 1997; Kennard and Woodruff-Pak, 2011; Kennard et al., 2013; Lopez-Otin et al., 2013; Kennedy et al., 2014). Of a variety of genetic and environmental factors that can influence the aging process, altered epigenetic landscapes have been well-documented during aging (Calvanese et al., 2009; Vlaming and Van Leeuwen, 2012; Huidobro et al., 2013; Booth and Brunet, 2016). Epigenetic modification is largely carried out by DNA methylation to reversibly regulate gene expression (Wu and Morris, 2001; Li, 2002; Suzuki and Bird, 2008), and DNA methylation levels were found to be associated with aging (Jones et al., 2015; Jung and Pfeifer, 2015). DNA methylation results in 5-hydroxymethylcytosine (5hmC), the first oxidized product of 5-methylcytosine (5mC), which is present in the mammalian genome and serves as an epigenetic indicator (Horvath, 2013). The 5hmC is enriched in the brain and undergoes dynamic change during neurodevelopment (Santiago et al., 2014). Moreover, an age-related increase of 5hmc levels has been described in the mouse brain (Szulwach et al., 2011). Since 5hmC is enriched in specific functional regions of the genome, such as the promoter and enhancer, it has been thought that 5hmC serves as a regulator in genetic transcription (Mellen et al., 2012). Nevertheless, the relationship between 5hmC modification and transcriptional regulation during brain aging has not been fully and rigorously investigated.

At present, human aging studies are largely based on a whole blood sample (Hannum et al., 2013; Johansson et al., 2013; Mcclay et al., 2014; Ciccarone et al., 2016; Kolodziej-Wojnar et al., 2020). This is because it is difficult to obtain human brain materials for investigation. As a result, limited studies were using human autopsy materials for the investigation of 5hmC in the human brain (Sjoholm et al., 2018; Jarmasz et al., 2019). However, variations in pre- and postmortem conditions affect the quality of collected tissues and can introduce confounds. Therefore, small animals, typically rodents, have been practically used in aging studies (Perrin, 2014). Although rodent brains are similar in structure and function to the human brain, the aging process between rodent and human brains is considerably different. For example, mice can live up to 3 years, which is much shorter than the life span (up to 100 years) of humans, Also, the early development of the brain is markedly different between rodents and primates (Azevedo et al., 2009; Lui et al., 2011; Workman et al., 2013; Otani et al., 2016). A noticeable structural difference is that rodent brains lack gyrification or the folding of the cortical surface, a unique structure seen in the brains of large animals. Thus, due to the evolutionary divergence between rodents and humans, it is important to use animals that are closer to humans to investigate the brain aging process. Rhesus monkey (*Macaca mulatta*) is one of the ideal models to investigate aging and diseases in the primates, given its closer similarities to humans in multiple aspects (Boffelli and Martin, 2012; Champagne, 2013; Cyranoski, 2016).

Here, we examined the 5hmC dynamics in the brain regions of rhesus monkeys at different ages. We found that 5hmC is accumulated in the brain in a tissue- and age-dependent manner. The dynamic changes of 5hmC during aging are likely associated with brain region-specific function and aging-related brain diseases. The findings in this study would help us to further explore how aging occurs in the primate brains and results in age-dependent neurological disorders.

## Materials and Methods

### Animal Tissues

The brain tissues of male rhesus monkeys at different ages were collected during our early studies of genetically modified monkey models (Yang et al., 2015, 2019, 2022; Yin et al., 2019). Three groups of rhesus monkeys (4 monkeys in each group) were used in the study (Supplementary Table 1). Specific brain regional tissues (cortex, cerebellum, hippocampus, and striatum) were dissected from euthanized rhesus monkeys, which were deeply anesthetized by intraperitoneal injection of 0.3–0.5 mg of atropine, followed by 10–12 mg of ketamine, and 15–20 mg of pelltobarbitalumnatricum per kg body weight. The dissected brain tissues were quickly saved in liquid nitrogen. All animal procedures were approved by the Institutional Animal Care and Use Committee at Guangdong Landao Biotechnology Co. Ltd, Guangzhou.

### Genomic DNA and RNA Isolation

Monkey brain tissues were homogenized on ice and then treated with proteinase K (0.667 μg μl−1) in digestion buffer (100 mM Tris-HCl (pH 8.5), 5 mM EDTA, 0.2% SDS (vol/vol), 200 mM NaCl) at 55 °C overnight. RNAse (4 mg) was then added for incubation at 37°C for 1 hour. An equal amount of phenol:chloroform: isoamyl alcohol (25:24:1, saturated with 10 mM Tris (pH 8.0) and 1 mM EDTA; P-3803, Sigma) was added to the tissue lysates to be mixed completely, followed by centrifugation for 10 min at 12,000 rpm. The aqueous layer was precipitated with an equal volume of isopropanol. The pellet was washed with 75% ethanol, air-dried, and resuspended with Nuclease-Free Water. Total RNA was purified from each tissue using Trizol (Invitrogen) according to the manufacturer's instructions. The concentration of DNA and RNA was measured by Qubit 3.0 (life Science).

### Dot Blot

Dot blot was performed on a Bio-Dot Apparatus (BIO-RAD) using rabbit antibody to 5-hmC (1:5,000, Active Motif) as the primary antibody, incubated overnight at 4°C. Horseradish peroxidase-conjugated antibody to rabbit (1:5,000, Sigma) was used as a secondary antibody and was incubated for 40 min at 20–25°C. Standard DNA templates were loaded (Zymo) for the quantification and for verification of the specificity of antibodies.

### 5hmC-Specific Enrichment

Genomic DNA was sonicated to ~200 bp by Bioruptor (each cycle with 30 s on and 30 s off, 13 cycles in total). The 5hmC enrichment was done, as previously described, with an improved selective chemical labeling method. The 5hmC labeling reactions were performed in a 30-μl solution containing 50 mM HEPES buffer (pH 7.9), 25 mM of MgCl_2_, 5–10 ug of sonicated genomic DNA, 100 μM of UDP-6-N3-Glu (Jena Bioscience), and 15 units of wild-type β-glucosyltransferase (New England Biolabs). Reactions were incubated for 1 h at 37°C. DNA substrates were purified *via* a Nucleotide removal kit (Qiagen) and reconstituted in H_2_O. About 4 ul of 1 mM DBCO-S-S-peg3-biotin was added into the DNA solution for incubation for 2 h at 37°C. Then, samples were purified by a DNA purification kit (Qiagen) following the manufacturer's recommendations. The biotin-labeled DNA was enriched by Streptavidin-coupled Dynabeads (Dynabeads® MyOne^TM^ Streptavidin T1, Life Technologies) and purified by a Qiagen Minelute purification kit.

### Library Preparation and High-Throughput Sequencing

The 5hmC-captured libraries were generated by the NEBNext® Ultra™ II DNA Library Prep Kit for Illumina according to the manufacturer's protocol. In short, 25 ng of input genomic DNA or 5hmC-captured DNA were used. The Agencourt AMPure XP beads (Beckman Coulter) were used for 200 bp DNA fragments selection after the adapter ligation step. An Agilent 2100 BioAnalyzer was used to quantify the amplified DNA. The final quality-insured libraries were sequenced on Illumina HiSeq X 10 (150bp paired-end reads) sequencer by Shenzhen Acegen Technology Co. Ltd.

### Quantification of Dot Blot

The density of each dot signal was quantified by ImageJ software. An unpaired *t*-test was used, and the data are presented as mean±SEM.

### Sequencing Read Quality Control and Alignment

All of the de-multiplexed sequencing reads, including 5hmC-captured libraries and RNA-seq libraries that passed filters, were first trimmed to remove the low-quality bases and adaptor sequences by Trimmomatic (ver. 0.36) using the following parameters: adapter.fa:2:30:7:1:TRUE; LEADING:3; SLIDINGWINDOW:4:15; TRAILING:3; and MINLEN:36. For the 5hmC-captured libraries, the trimmed reads were uniquely mapped to reference genome rheMac8 by BWA, with default parameters. After filtering the duplicate reads, the mapping information for each read pair was extracted and output as a file in BED format for the following analysis.

### Visualization of Reading Densities

We used normalized reads number per 50 bp among the genome of the 5hmC-captured sequence reads to calculate the read density normalized to one million reads in the library for each genomic position (Wig files). Screenshots of genomic regions were taken using the Integrative Genomics Viewer (IGV) genome browser.

### Identification of Enriched Regions and Tissue- and Age-Specific DhMRs

Regions enriched with 5hmC (also known as peaks) over the corresponding input of each sample were identified using the peak calling software MACS (version 1.4.9) with default parameters. PePr (version 1.1.1.18) was used for tissue- and age-specific DhMRs detection, which uses a sliding window approach and models read counts across replicates and between groups with a negative binomial distribution (Zhang et al., 2014). Venn diagram of genes with hypermethylated DhMRs and hypomethylated DhMRs was achieved by R software for data statistics and graphing. DhMR genes related to cerebellar diseases were analyzed using the Cytoscape platform.

### Sequential Features Analysis

To identify age-specific 5hmC-modified genes, or regions with age in different brain tissues, fuzzy clustering (implemented by the mfuzz package of R software) was used. Each cluster was obtained by clustering DhMR at a specific age, and function enrichment analysis of each cluster-related gene was performed.

#### Cluster Dendrograms

The human and mouse hydroxymethylation data sets (GEO: GSE32188) in the NCBI GEO database were downloaded according to the published data for 5-hmC-mediated epigenetic dynamics during postnatal neurodevelopment and aging. Referring to the data processing method of rhesus monkeys, the downloaded data is initially quality-controlled to remove low-quality reads containing linkers; then, the clean data is compared to the human (hg19) and mouse (mm10) genomes to obtain sequencing reads. Determined homologous genes among humans, monkeys, and mice were standardized by the RMP method [RPM = Total gene reads/ Mapped reads (Millions)] to facilitate comparison. The sva package of R software was used to remove the deviation caused by batches. Finally, we used UPGMA (Unweighted Pair-group Method with Arithmetic Mean) to perform cluster analysis of samples and draw cluster dendrograms.

## Results

### Aging-Dependent 5hmC Accumulation in Four Brain Regions of Rhesus Monkey

**Figure 1:**
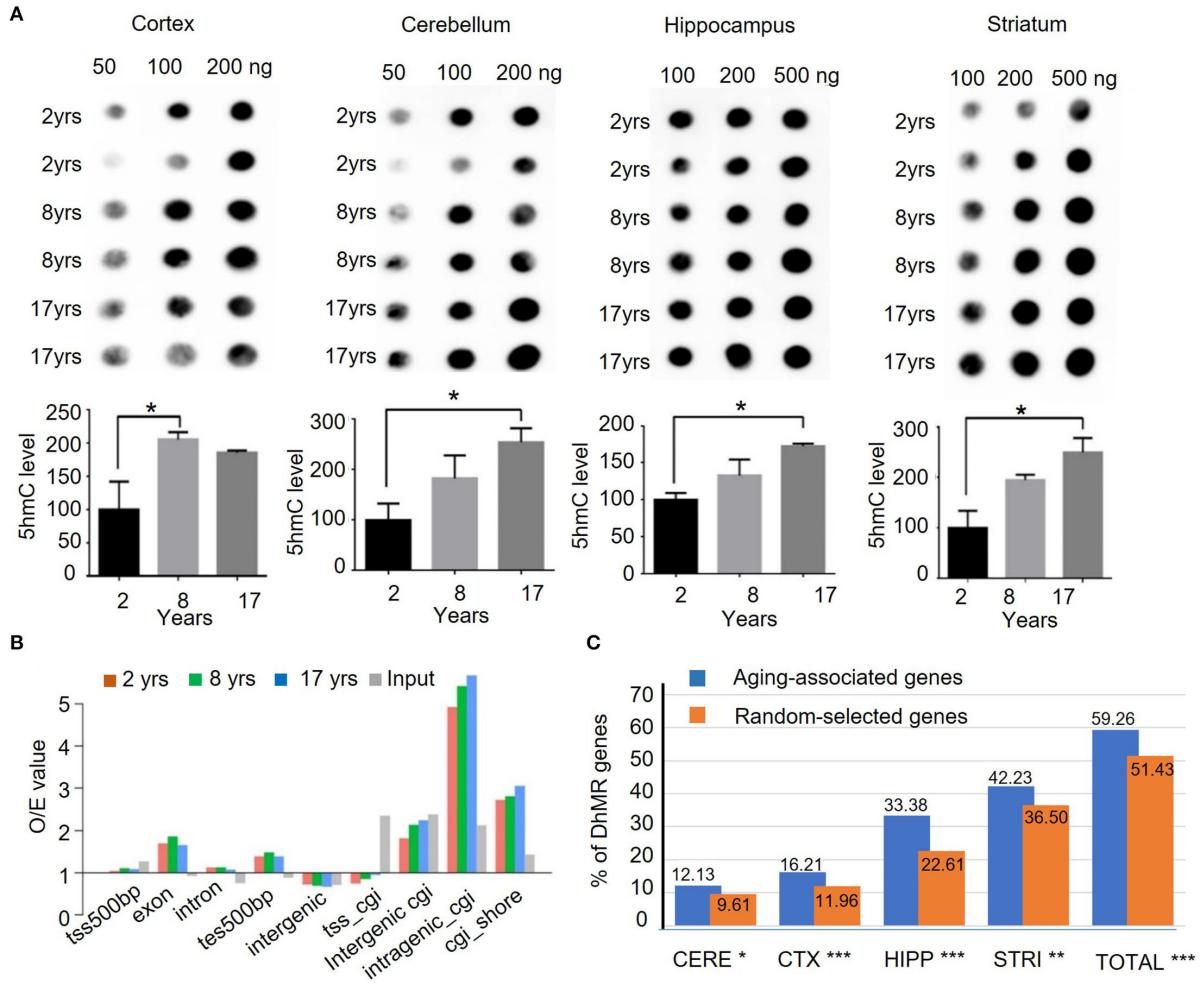
Quantification and genomic mapping of 5hmC in four brain regions in rhesus monkeys. **(A)** 5hmC-specific dot blot revealed accumulation of 5hmC in cortex, cerebellum, hippocampus, and striatum of rhesus monkeys at 2, 8, and 17 years. Quantification of 5hmC-specific immunoblot by imageJ software in cortex, cerebellum, hippocampus, and striatum of rhesus monkeys. (*n* = 2, *p < 0.05; unpaired *t-*test, error bar = mean±SEM). **(B)** 5hmC distribution at different genomic components in 2-, 8-, and 17-year-old groups. **(C)** The ratio of genes with DhMRs to aging-associated genes (734) in different brain regions and the combined regions (totally). *p < 0.05, **p < 0.01, ***p < 0.001; unpaired *t-*test.

Although the 5hmC level was found to undergo dynamic change through development and aging in mice (Szulwach et al., 2011), it has not been defined in different brain regions in human and non-human primates. To profile the global changes in 5hmC modification during brain aging, genomic DNA was isolated from 4 brain regional tissues (the cortex, cerebellum, hippocampus, and striatum) of rhesus monkeys at 3 different ages: 2 years (juvenile), 8 years (young adult, named young), and 17 years (old). These brain tissues were freshly collected and well-preserved in liquid nitrogen. For investigating 5mhC in the brain genomics, each age group (juvenile, young, old) consisted of 4 monkeys, and the genomic DNAs from 4 different brain regions (the cortex, cerebellum, hippocampus, and striatum) were isolated. Figure 1A shows representative 5hmC-specific dot-blotting of brain DNA samples. Quantification of dot blots showed a significant accumulation of 5hmC levels in these 4 brain regions as age increased (*P* < 0.05, Figure 1A). The 5hmC in the cerebellum and striatum of rhesus monkeys showed high signals, which are consistent with the findings in the mouse and human cerebellums (Li and Liu, 2011; Szulwach et al., 2011). The increased levels of 5hmC in the monkey brains from young to old age indicate that an age-dependent increase of 5hmC is conserved in mammalian species.

### Genomic Features of 5hmC During Aging

To assess genome-wide 5hmC distribution in different brain regions during aging, the aforementioned genomic DNA was fragmented, and then, captured *via* chemical labeling, followed by affinity 5hmC-enrichment for deep sequencing. The total genomic DNA was used as an input and was sequenced at the same time. The total reads for all samples were >30 million per sample, Q20 > 94%, and Q30 > 87% (Supplementary Table 2), and the uniquely mapped reads rate reached 93.74% (Supplementary Figure 1A). Processed data were mapped onto the rhesus monkey genome (rheMac2) using Burrows-Wheeler Aligner software, and the mapping ratio of each sample reached over >95%. The 5hmC enrichment intensity was analyzed using binned data. Read numbers were calculated at each bin, from 100 bins at 2 kilobases of upstream or downstream with transcriptional starting site (TSS) and transcriptional ending site (TES). Analysis of the genomic features of the dynamic 5hmC regions showed that 5hmC was concentrated on intragenic CpG islands (GGIs, more than 5-fold than expected), intergenic CGIs, and CGIs shores (±2kb of CGIs), whereas little enrichment of 5hmC was found on exons with ±500bp of TES (Figure 1B). Chromosomal 5hmC densities analysis identified dynamic 5hmC regions over the genome. Of note, we observed that the distribution of 5hmC on chromosomes 17, 18, and X was lower than on other chromosomes, whereas it was depleted on chromosome Y (Supplementary Figure 1B) in all four tissues and at all ages. Since DNA methylation on repetitive elements was found to be associated with genomic stability and is implicated in neuronal development (Coufal et al., 2009; Bollati et al., 2011; Putiri and Robertson, 2011), we examined the genome-wide distribution of 5hmC on repetitive elements by aligning the 5hmC reads to the RepeatMasker annotated portion of the genome directly. In all four brain regions, 5hmC signals of short interspersed nuclear element (SINE) were significantly enriched when compared with input. The enrichment of 5hmC on SINE was even more significant in the striatum of the old monkey group (25.8% increase from juvenile to old). By contrast, 5hmC marking of low complexity repeats was comparable to unenriched genomic DNA in these 4 brain regions (Supplementary Figure 1C). Overall, these results suggest the age-dependent accumulation and region-specific distribution of 5hmC in the brain of the rhesus monkey.

We employed a Poisson-based peak identification algorithm (MACS) and identified 13,600 genes with differentially hydroxymethylated regions (DhMRs). Based on published data (Hannum et al., 2013; Horvath, 2013; Johansson et al., 2013; Mcclay et al., 2014) and online database (https://genomics.senescence.info/genes/index.html), we identified 734 genes that are associated with aging (Supplementary Table 3). Of these 734 genes, 435 (59.26%) genes have DhMRs at a higher level than randomly selected genes. Among 4 monkey brain regions, the hippocampus and striatum showed a closer correlation between DhMR and aging-associated genes (Figure 1C), supporting the notion that dynamic 5hmC change is correlated with aging in a brain region-dependent manner.

### Unique 5hmC Pattern in the Cerebellum

**Figure 2:**
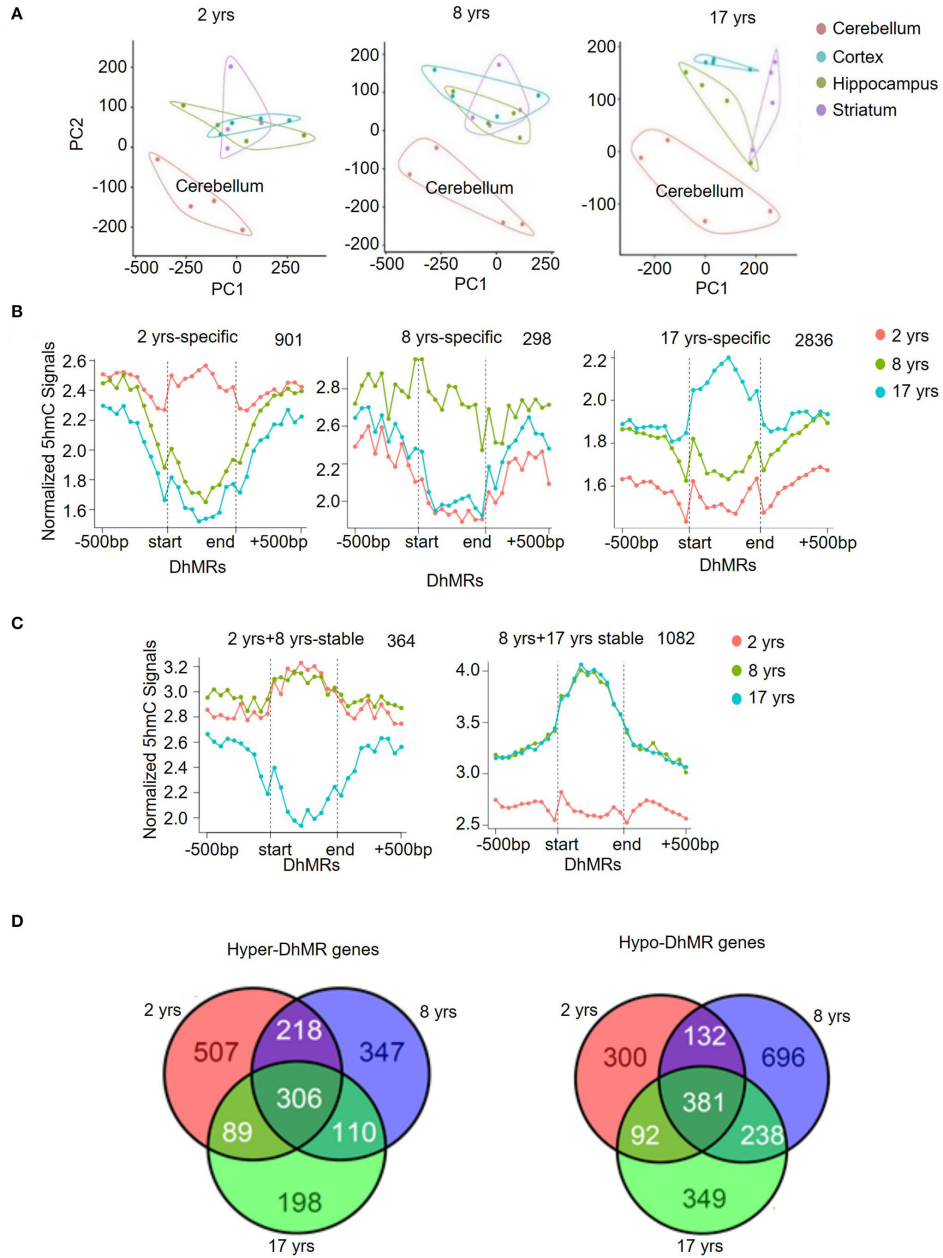
Unique 5hmC pattern in the cerebellum. **(A)** PCA result of all samples. **(B)** PCA results for 2-, 8-, and 17-year-old monkeys. **(C)** Identification and characterization of cerebellum-specific dynamic DhMRs. A total of 901 DhMRs identified as juvenile specific relative to young and old groups, 298 DhMRs identified as young specific relative to juvenile and old groups, and 2,836 DhMRs identified as old specific relative to juvenile and young groups. **(D)** Identification and characterization of cerebellum-specific stable DhMRs. A total of 364 DhMRs were identified as juvenile and young stable relative to the old group and 1,082 DhMRs were identified as young and old stable relative to the juvenile group. The total numbers of DhMRs are indicated in the upper right-hand corner of each figure.

Dot-blotting and quantification results suggested that 5hmC was accumulated in the brain, but its abundance varied among brain regions. To better understand the 5hmc enrichment pattern in the monkey brain, we used principal component analysis (PCA) to infer the inter-group global patterns of 5hmC, based on 5hmC levels for 1-kb tiles across the whole genome from 48 diverse brain tissues. Strikingly, the global 5hmC in the cerebellum displayed a conspicuous grouping that was completely separated from the cortex, hippocampus, and striatum at all ages (Figure 2A). The 5hmC groupings of the cortex, hippocampus, and striatum were overlapped at the juvenile and young ages, but became distinctly distributed in the old monkey brain, suggesting differential regulatory roles of 5hmc in these three brain regions when animals became old. Together, these data demonstrated a dynamic change of 5hmC in an age-dependent manner.

To further analyze the genomic 5hmC characteristics in brain tissues during aging, we examined tissue- and age-specific DhMRs using a Poisson-based method (Zhang et al., 2008). We identified 99,431 tissue-specific DhMRs (Supplementary Table 4) and 68,481 age-specific DhMRs (Supplementary Table 5). Similar distribution patterns of 5hmC DhMRs were seen in the monkey hippocampus (Supplementary Figure 2A) and cortex (Supplementary Figure 2B) except for the 8-year-old group. However, cerebellum-specific DhMRs distributions were more distinct and concentrated on intragenic and intergenic CpG islands, as well as in exon (Supplementary Figure 3A). The DhMRs enrichment on these three DNA regions was stable at all ages. Compared to the other three brain regions combined, the cerebellum showed 29,743 DhMRs in the juvenile group, 20,151 DhMRs in the young group, and 15,749 DhMRs in the old group (Supplementary Figure 3B), which was defined as a gain of DhMRs in the cerebellum. As a comparison, the other three brain regions altogether showed 10,394 DhMRs in the juvenile group, 14,027 DhMRs in the young group, and 9,367 DhMRs in the old group (Supplementary Figure 3C), which were defined as a loss of 5hmC in the cerebellum. In accordance with the PCA analysis, these data demonstrated selective 5hmC enrichment in the cerebellum across all age groups.

Modification of 5hmC, acting as a stable DNA mark, has been revealed by multiple studies (Hahn et al., 2014; Hu et al., 2015). We investigated this concept in the context of rhesus monkeys during aging. We categorized DhMRs in each brain region into 3 age-specific groups (juvenile-, young- and old-specific) (Figure 2B) and stable groups across 2 ages (juvenile+young-stable and young+old-stable) (Figure 2C). The numbers of specific and stable DhMRs were summarized in Supplementary Table 5. The 364 DhMRs were stably maintained from juvenile to young monkeys, and 1,082 stable DhMRs in young and old monkeys (Figure 2C). These data suggested that 5hmC was stably maintained in the monkey brain with age. As a comparison, 901,298 and 2,836 age-specific DhMRs have been found in the juvenile, young, and old groups (Figure 2B). The greatest number of DhMRs was found in the cerebellum of old monkeys. We also analyzed hypermethylated and hypomethylated DhMRs. In total, 1,775 genes with hypermethylated DhMRs and 2,188 genes with hypomethylated DhMRs were identified in the cerebellum. Among them, 306 hyper-DhMRs and 381 hypo-DhMRs were found across all age groups (Figure 2D).

### Association of DhMRs in the Old Monkey Striatum With Learning and Locomotory Behavior Pathways

**Figure 3:**
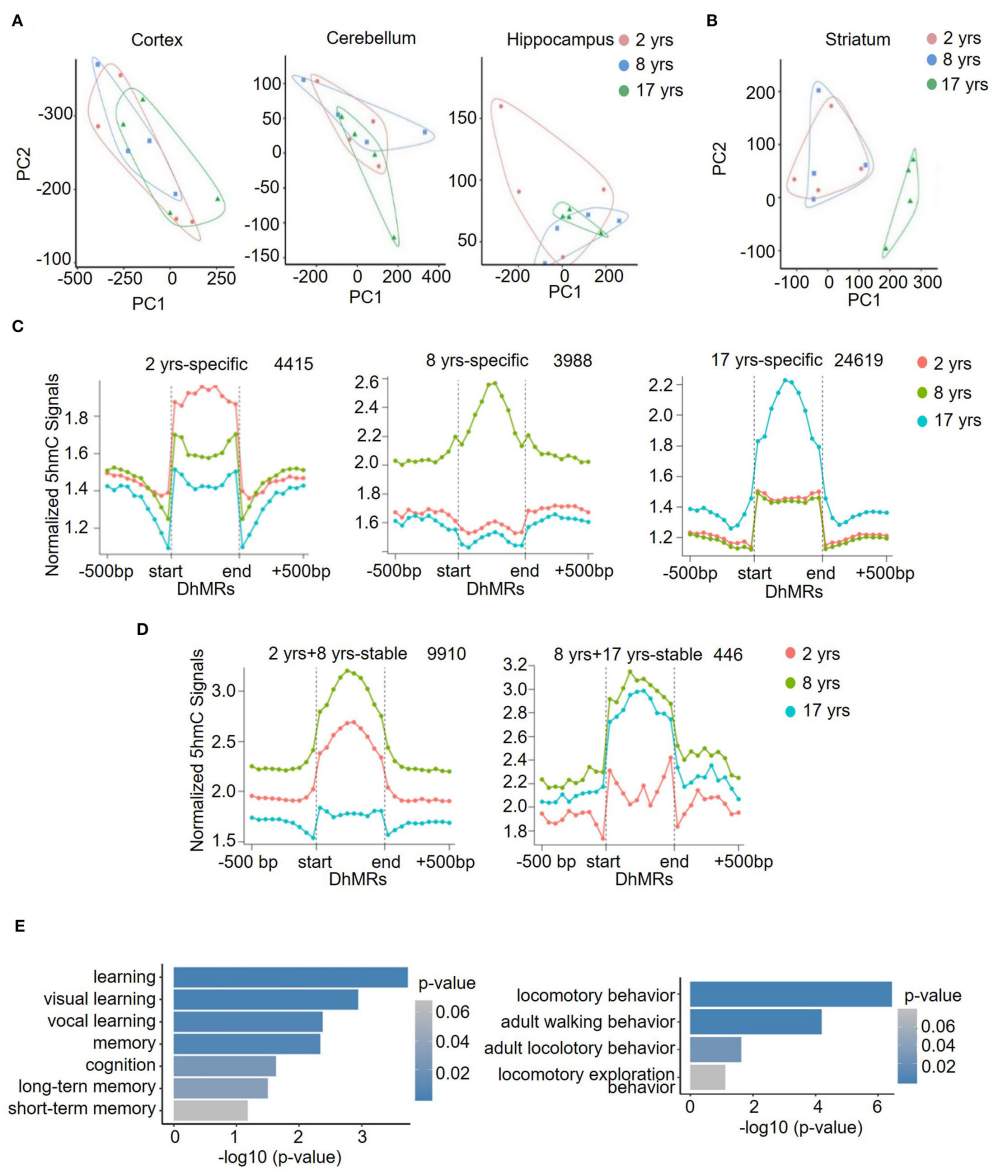
Identification and characterization of striatum-specific DhMRs. Principal component analysis (PCA) results in the cortex, cerebellum, hippocampus **(A)**, and striatum **(B)**. **(C)** Dynamic 5hmC DhMRs in the striatum. A total of 4415 DhMRs were identified as 2 years-specific relatives to 8 years and 17 years groups, 3,988 DhMRs were identified as 8 years-specific relatives to 2 years and 17 years groups, and 24,619 DhMRs were identified as 17 years-specific relatives to 2 years and 8 years groups. **(D)** Stable 5hmC DhMRs in the striatum. A total of 9,910 DhMRs were identified as 2 years and 8 years stable relative to the 17 years group, and 446 DhMRs were identified as 8 years and 17 years stable relative to the 2 years group. The total numbers of DhMRs are indicated in the upper right-hand corner of each figure. **(E)** GO terms of 17-years-specific DhMRs enriched in cognition (left) and locomotory behavior (right) pathways. The y-axis lists the GO terms, and the x-axis indicates the significance *p*-value of enrichment.

Further PCA analysis demonstrated that in contrast to the comparable global 5hmC patterns of cortex, cerebellum, and hippocampus across different age groups (Figure 3A), a separated grouping was present in the striatum of the old group (Figure 3B). Moreover, in all four brain regions at all ages, the striatum in the old group possessed the highest number of DhMRs (Figures 3C,D), where a total of 24,619 DhMRs were identified (Figure 3C). The striatum is important for controlling cognition, reward processes, and movement coordination (Simpson et al., 2010; Baez-Mendoza and Schultz, 2013; Tewari et al., 2016), and the dysfunction of the striatum contributes to a variety of diseases, such as depression, schizophrenia, Parkinson's disease (PD), and Huntington's disease (HD) (Subramaniam and Snyder, 2011; Lieu and Subramanian, 2012; Jiang et al., 2019; Mccutcheon et al., 2019). To assess the functional significance of this unique 5hmC enrichment in aged striatum, GO analyses were conducted to determine the involvement of 5hmC in striatum-related functions. The DhMRs of the old monkey striatum were found to be significantly enriched in learning and locomotory behavior pathways (Figure 3E, Supplementary Table 6). These data suggested that 5hmC may be involved in normal aging of the striatum through the regulation of key genes related to the process of cognitive control and motor functions.

### DhMRs Clustering Displayed a Tissue-Specific Pattern

**Figure 4:**
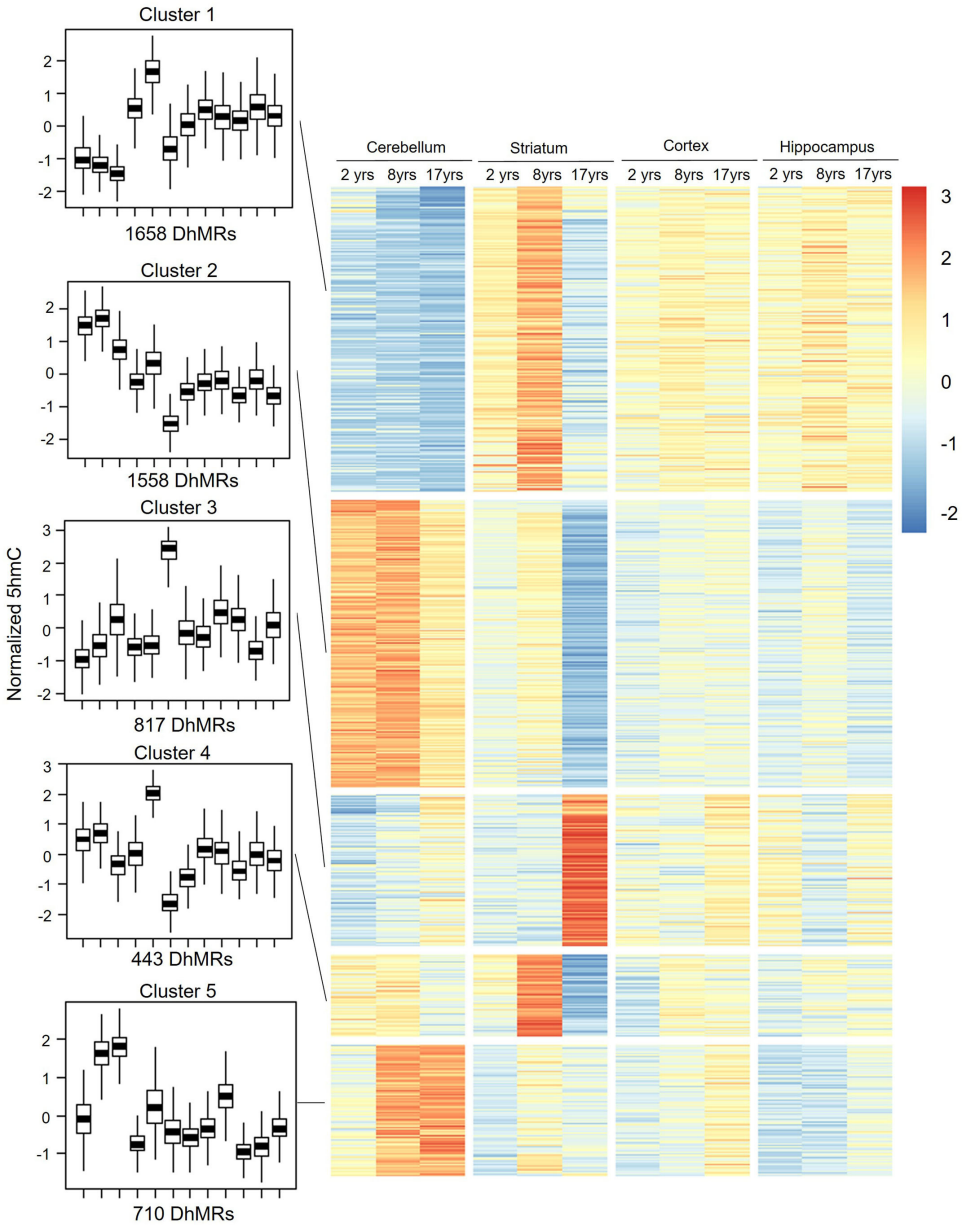
DhMRs clustering. Five clusters were grouped according to significant differences, which consist of 1,658 DhMRs in Cluster 1, 1,558 DhMRs in Cluster 2, 817 DhMRs in Cluster 3, 433 DhMRs in Cluster 4, and 710 DhMRs in Cluster 5.

**Figure 5:**
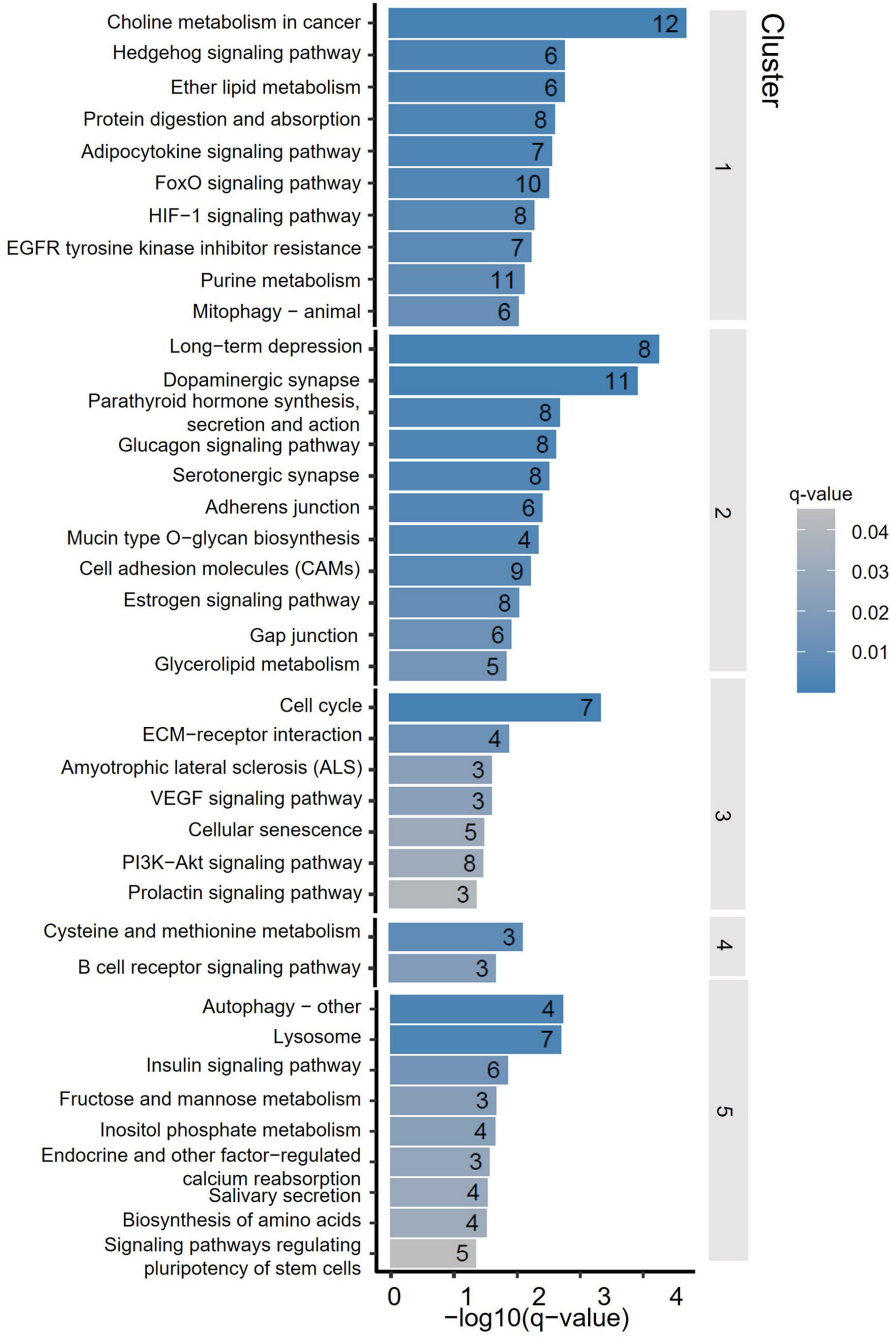
Kyoto Encyclopedia of Genes and Genomes (KEGG) terms of DhMRs from 5 clusters. The y-axis lists the KEGG terms, and the x-axis indicated the significance q-value of enrichment.

To assess the distribution feature of DhMRs in all tissues and ages, we grouped the DhMRs into 5 clusters with different features (Figure 4). Notably, cluster 1 (1,658 DhMRs) showed lower 5hmC signals in all three age stages of the cerebellum, but higher in juvenile and young groups of the striatum. Importantly, Kyoto Encyclopedia of Genes and Genomes (KEGG) analyses revealed that particularly and significantly enriched pathways of cluster 1 (top 10 terms based on the significant difference) were related to signal transduction and metabolism (Figure 5). Oppositely, cluster 2 (1,558 DhMRs) showed relatively high signals of 5hmC in all three age stages of the cerebellum rather than in other tissues. The enriched pathways were correlated with the nervous system and endocrine system, suggesting that higher 5hmC is involved in the normal function of the cerebellum, such as synapse function and long-term depression. It is noteworthy that the striatum displayed significantly high 5hmC patterns in the old group (cluster 3, 817 DhMRs) and young group (cluster 4,443 DhMRs), and these age-specific correlative genes were involved in signaling transduction and metabolism pathways (Figures 4, 5). Overall, these results implied that tissue-specific 5hmC patterns may have a potential regulation of gene expression in a tissue-specific manner.

### Sequential Features of 5hmC DhMRs in Different Regions of the Monkey Brain During Aging

**Figure 6:**
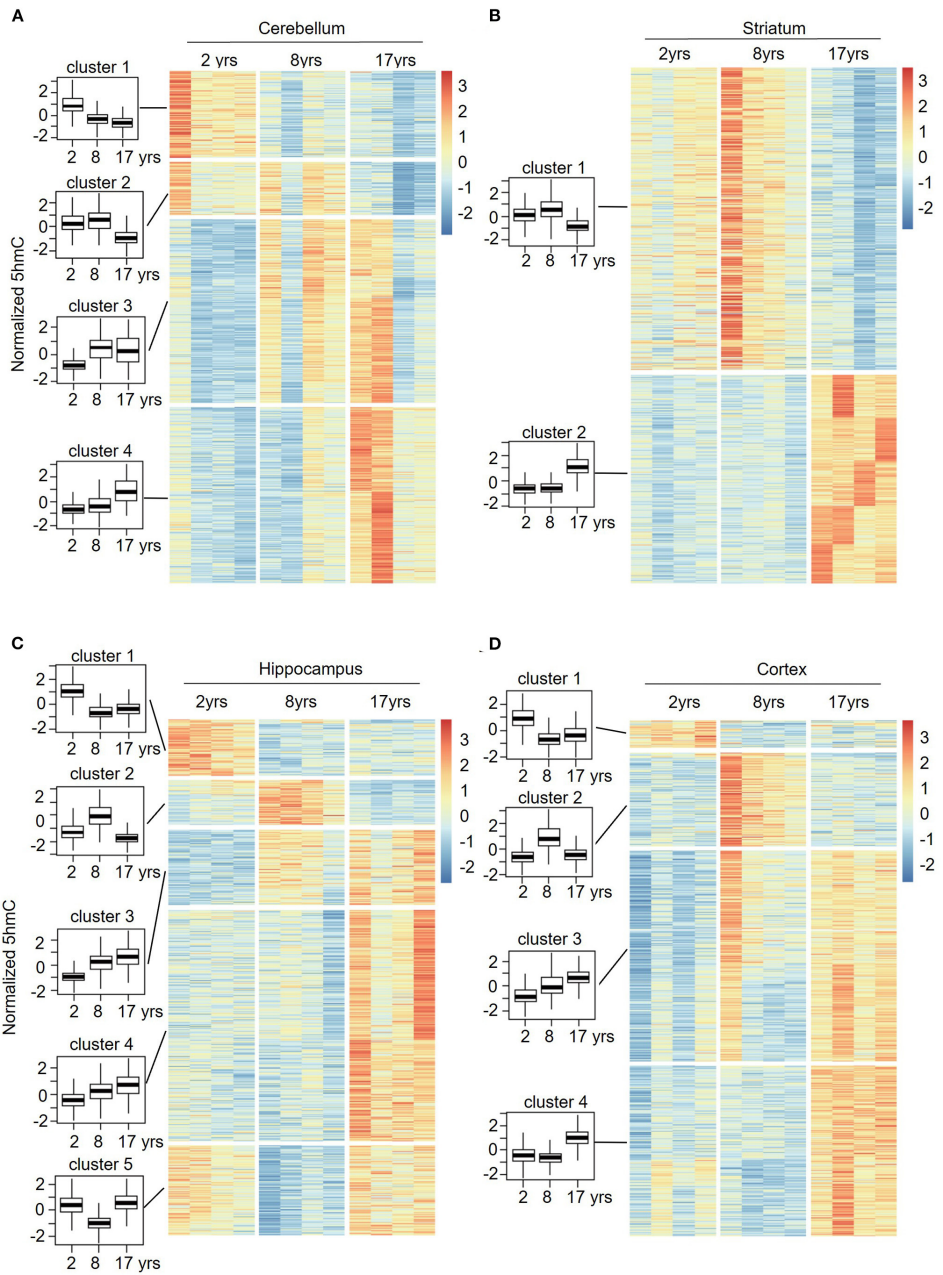
Sequential features of 5hmC at cerebellum **(A)**, striatum **(B)**, hippocampus **(C)**, and cortex **(D)** during aging. Time-series analysis revealed 4 clusters in the cerebellum **(A)**, 2 clusters in the striatum **(B)**, 5 clusters in the hippocampus **(C)**, and 4 clusters in the cortex **(D)** from young to old.

To understand the change patterns of 5hmC during aging, we analyzed the sequential features of DhMRs across different age groups using fuzzy clustering (R software mfuzz package) (Figures 6A–D). KEGG analyses of each set of cluster-related genes were then performed. Four sets of changed clusters in the cerebellum (clusters 1-4) were obtained: clusters 1 and 2 with lower-level 5hmC in the old monkeys, and clusters 3 and 4 with lower-level 5hmC in the juvenile monkeys, whereas differential 5hmC levels were found in the young monkeys (Figure 6A). KEGG analyses of clusters 1 and 2 revealed the enrichment of biological pathways that are involved in neuronal function and signal transduction (Supplementary Figure 4A), suggesting a regulatory role of 5hmC in cerebellum development. Interestingly, the KEGG analyses of clusters 3 and 4 showed increased 5hmC levels in the aging-related pathway (longevity regulation pathways) (Supplementary Figure 4A). In the striatum, we obtained 2 sets of changed clusters: cluster 1 with lower-level 5hmC in the old group and cluster 2 with higher-level 5hmC in the old group (Figure 6B). KEGG analyses of both groups indicated that enriched genes were implicated in neuronal function, signal transduction, and cellular communication (Supplementary Figure 4B). Together, the sequential features of 5hmC in the cerebellum and striatum indicated that the changes of 5hmC are tightly related to neuronal development and signal transduction during aging. Noticeably, the longevity regulation pathways were enriched in clusters 1 and 3 of the cerebellum (Supplementary Figure 5A), clusters 1 and 2 of the striatum (Supplementary Figure 4B), clusters 4 and 5 of the hippocampus (Supplementary Figure 5A), and cluster 3 and 4 of the cortex (Supplementary Figure 5B). These data indicated that the dynamic 5hmC modifications were involved in neuronal development and associated with longevity regulation.

### 5hmC Patterns in the Brains of Different Species

**Figure 7:**
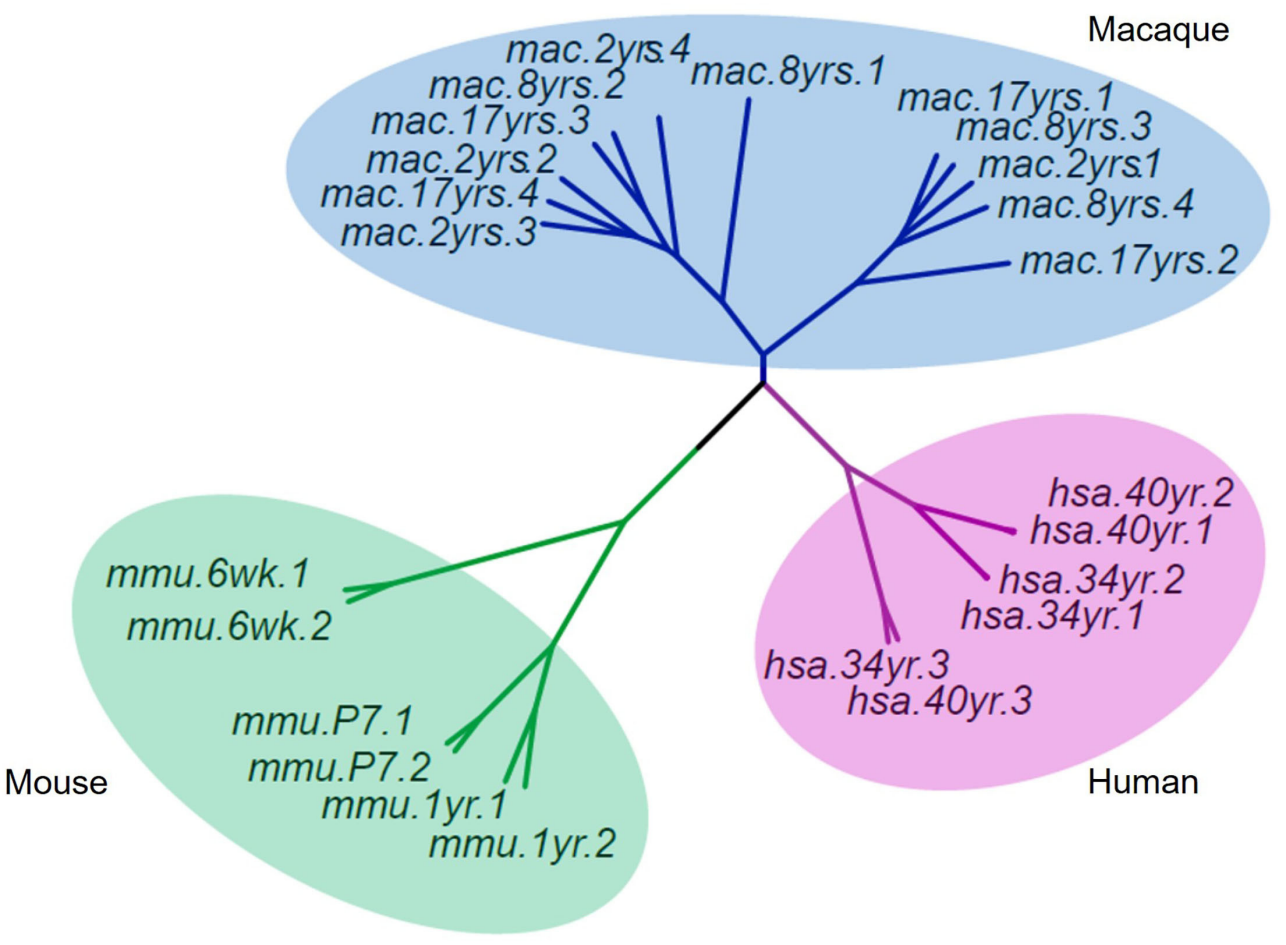
Cluster dendrograms of 5hmC among rhesus, human, and mouse. The 5hmC pattern of the rhesus monkey was closer to that of humans than mice.

To understand the evolutionary importance of enriched DhMRs in the rhesus monkey brain, we compared the 5hmC pattern of monkey's cerebellum to that of humans' and mice's from published studies (Szulwach et al., 2011). Based on gene function annotation, 14,526 homologous genes were identified among humans, monkeys, and mice. Using the sva package of R language to remove the deviation caused by batches, the standardized RMP (RPM = total gene reads/ mapped reads (millions)) matrix of the 5hmC performance cluster was processed by UPGMA (Unweighted Pair-group Method with Arithmetic Mean) to construct cluster dendrograms. The phyllo-epigenetic analyses revealed that, compared to mice, the 5hmC features of rhesus monkeys are closer to those of humans (Figure 7). These results indicated that non-human primates, such as the rhesus monkey, are better animal models to study epigenetic regulation related to human brain function and aging.

## Discussion

The 5hmC is considered the sixth (5mC is the fifth) base in the mammalian genome that plays a role in the development and aging (Song and He, 2011; Mellen et al., 2012). Here, we investigated the distribution of 5hmC in different brain regions of rhesus monkeys at different ages through integrative analysis of genome-wide 5hmC. We observed an overall accumulation of 5hmC in all four brain regions as age increases. Moreover, the cerebellum displayed a unique pattern of 5hmC modification across all age groups, and the striatum shows a specific alteration of 5hmC in old monkeys. Further analysis showed that the brain region-specific DhMRs were enriched in neuronal function and signal transduction pathways that are associated with brain region-specific function and aging-related brain diseases. Together, these data suggest that dynamic 5hmC is involved in the aging process of the primate brains and may play a role in the pathogenesis of aging-related brain diseases.

Horvath et al. first proposed that the degree of 5mC at specific sites in DNA can be used to predict biological age, and DNA methylation (5mC) has been called the epigenetic clock (Horvath, 2013). The ten-eleven translocation (Tet) proteins catalyze the oxidation of 5mC to form 5hmC (Tahiliani et al., 2009; Ito et al., 2010), and 5hmC is the first oxidative product in the active demethylation of 5mC. Therefore, the pattern of 5hmC alterations should closely reflect the change in 5mC and has been used as an indicator of the epigenetic clock to estimate ages. Consistent with this theory, 5hmC is markedly increased from the early postnatal stage to adulthood in the cerebellum and hippocampus of mice (Szulwach et al., 2011; Chen et al., 2012). A previous study of frozen human tissues also showed that 5hmC has higher signals in the human brain than peripheral tissues (Li and Liu, 2011) and is accumulated in aged cells (Borkowska et al., 2020). However, the brain regional changes of 5hmC during primate aging have not been fully investigated. Our data demonstrated that, in non-human primates' brains, the accumulation of 5hmC also increased with age, suggesting that the increase of 5hmC with age is conserved across different species. The novel findings in our study are that genomic 5hmC accumulation in the primate brain is brain region- and age-dependent. The non-uniform distribution of 5hmC in the whole genome is also supported by our finding that the density of 5hmC on the X chromosome was lower than euchromosome, and the 5hmC on the Y chromosome was depleted. Further, we found that tissue-specific DhMRs were enriched on CpG islands and exons. The brain region- and age-dependent distribution of 5hmC in the whole genome may play a regulatory role in brain region-specific function and aging-related diseases.

Previous studies have shown that the rate of senescence differs among tissues. The mouse cerebellum shows earlier senescence than the hippocampus, but the human cerebellum ages more slowly than other parts of the human body (Woodruff-Pak et al., 2010; Horvath et al., 2015). In our study, we found that the 5hmC displayed a specific pattern in the cerebellum and that cerebellum-specific DhMRs were involved in neuronal function and signal transduction. These results implied that a unique 5hmC pattern may be associated with the rate of aging through regulation of neuronal development and signal transduction pathways in the cerebellum. The cerebellum has been considered to play roles in coordination, locomotion (Pisotta and Molinari, 2014; Macri et al., 2019), and cognition (Schmahmann, 1998, 2004; Buckner, 2013; Elkiss, 2020), and its dysfunction is involved in cognitive dysmetria and disequilibrium (Andreasen and Pierson, 2008; Mothersill et al., 2016), and several neurodegenerative diseases with ataxia symptoms that are typically characterized by a lack of muscle control or coordination of voluntary movements (Manto and Marmolino, 2009; Smeets and Verbeek, 2014). Our results showed that the cerebellum-specific 5hmC patterns might provide new insights into the pathogenesis of cerebellum-related neurodegenerative diseases. The age-dependent 5hmC distribution in the striatum is also likely to be associated with an age-dependent decline in neuronal function and signal transduction that contributes to the selective vulnerability of striatal neurons in age-dependent neurodegenerative diseases, such as Huntington's disease (Bates et al., 2015).

Phylogenetically, rhesus monkeys are closer to humans than other species, with about 93% similarity in DNA sequences (Rhesus Macaque Genome et al., 2007; Cyranoski, 2016). Using an array-based assay of whole-brain tissues, previous studies revealed that the 5mC and 5hmC showed similar genomic profiles between humans and monkeys and that the species-specific distributions of 5mc and 5hmC were enriched on genes associated with the neuronal function (Chopra et al., 2014; Madrid et al., 2018). However, it remains unknown how 5hmC is distributed in different human brain regions at different ages. In our studies, we used freshly collected and well-preserved primate brain tissues to investigate age-dependent 5hmC accumulation and enrichment. Our data convincingly showed that 5hmC modifications were enhanced in the rhesus monkey brain during aging. Importantly, with the high quality of monkey brain tissues, we were able to identify, for the first time, the cerebellum-specific 5hmC pattern and age-dependent 5hmC change in the striatum, which have not been reported in the previous studies of a mouse or human tissues. The association of the brain region- and age-related DhMRs with neuronal function supports the functional implication of these changes for a brain-region specific function and potentially aging-related diseases. These findings will help us understand the mechanism underlying the brain aging process and age-dependent brain diseases in humans.

Our findings also suggest that it would be important to investigate 5-methylcytosine (5mC), which can be converted to 5hmC by TET families. Both 5mC and 5hmC could regulate gene expression during development and aging. Further analyses of the relationship between 5mC and 5hmC in the rhesus monkeys will provide a clearer picture of how different methylation modifications integratively regulate gene expression during brain aging.

## Data Availability Statement

The datasets presented in this study can be found in online repositories. The names of the repository/repositories and accession number(s) can be found below: https://www.ncbi.nlm.nih.gov/bioproject/PRJNA688531.

## Ethics Statement

The animal study was reviewed and approved by Institutional Animal Care and Use Committee at Guangdong Landao Biotechnology Co. Ltd, Guangzhou.

## Author Contributions

LL and X-JL conceived the project. LL, YX, LZ, and HW performed experiments. YL, JX, and LX processed and managed sequencing data and performed statistical analysis. XG, PY, and XC collected and provided monkey tissues. LL, JZ, SL, and X-JL interpreted results and drafted the manuscript. All authors edited and approved the final manuscript.

## Funding

This work was supported by grants from the Natural Science Foundation of Guangdong Province (2019A1515011416 and 2022A1515010689), National Key Research and Development Program of China Stem Cell and Translational Research (2017YFA0105102), Key Field Research and Development Program of Guangdong province (2018B030337001), Guangzhou Key Research Program on Brain Science (202007030008), and Guangdong Key Laboratory of non-human primate models of brain diseases.

## Conflict of Interest

The authors declare that the research was conducted in the absence of any commercial or financial relationships that could be construed as a potential conflict of interest.

## Publisher's Note

All claims expressed in this article are solely those of the authors and do not necessarily represent those of their affiliated organizations, or those of the publisher, the editors and the reviewers. Any product that may be evaluated in this article, or claim that may be made by its manufacturer, is not guaranteed or endorsed by the publisher.
