# Supplementary material for: Brain Region- and Age-Dependent 5-Hydroxymethylcytosine Activity in the Non-Human Primate
Source: Front Aging Neurosci. 2022 Jul 13;14:934224. doi: 10.3389/fnagi.2022.934224 (PMC9326314; doi:10.3389/fnagi.2022.934224)
Supplement: Supplementary file 1 [file Data_Sheet_1.docx]

**Supplementary Table 1. Rhesus monkeys' information in the study.**

| **Group** | **Number** | **Age** | **Sex** |
| --- | --- | --- | --- |
| 2 years  (Juvenile) | 2yrs-1 | 2 | Male |
|  | 2yrs-2 | 2 | Male |
|  | 2yrs-3 | 2 | Male |
|  | 2yrs-4 | 2 | Male |
| 8 years  (Young) | 8yrs-1 | 8 | Male |
|  | 8yrs-2 | 8 | Male |
|  | 8yrs-3 | 8 | Male |
|  | 8yrs-4 | 8 | Male |
| 17 years  (old) | 17yrs-1 | 17 | Male |
|  | 17yrs-2 | 17 | Male |
|  | 17yrs-3 | 17 | Male |
|  | 17yrs-4 | 17 | Male |

**Supplementary Table 2. Summary of 5hmC sequencing data.**

| **Sample** | **Raw_base** | **Clean_reads** | **Clean_base** | **Clean_rate** | **Q20_base** | **Q20_rate** | **Q30_base** | **Q30_rate** |
| --- | --- | --- | --- | --- | --- | --- | --- | --- |
| 2 yrs-1-CERE | 1.07E+10 | 70851750 | 1.07E+10 | 99.97% | 1.02E+10 | 95.23% | 9.57E+09 | 89.46% |
| 2 yrs-1-CTX | 1.36E+10 | 90214722 | 1.36E+10 | 99.97% | 1.29E+10 | 94.95% | 1.21E+10 | 89.10% |
| 2 yrs-1-HIPP | 1.41E+10 | 93467390 | 1.41E+10 | 99.97% | 1.34E+10 | 95.08% | 1.26E+10 | 89.32% |
| 2 yrs-1-STRI | 4.14E+09 | 27402364 | 4.14E+09 | 99.97% | 3.92E+09 | 94.71% | 3.67E+09 | 88.65% |
| 2 yrs-2-CERE | 8.87E+09 | 58749074 | 8.87E+09 | 99.97% | 8.43E+09 | 95.00% | 7.9E+09 | 89.03% |
| 2 yrs-2-CTX | 1.22E+10 | 81077522 | 1.22E+10 | 99.97% | 1.16E+10 | 94.93% | 1.09E+10 | 89.13% |
| 2 yrs-2-HIPP | 7.58E+09 | 50152126 | 7.57E+09 | 99.97% | 7.15E+09 | 94.39% | 6.68E+09 | 88.20% |
| 2 yrs-2-STRI | 1.07E+10 | 70705418 | 1.07E+10 | 99.97% | 1.02E+10 | 95.40% | 9.6E+09 | 89.91% |
| 2 yrs-3-CERE | 1.16E+10 | 76747240 | 1.16E+10 | 99.98% | 1.11E+10 | 96.12% | 1.05E+10 | 90.80% |
| 2 yrs-3-CTX | 1.02E+10 | 67563586 | 1.02E+10 | 99.98% | 9.8E+09 | 96.04% | 9.26E+09 | 90.73% |
| 2 yrs-3-HIPP | 9.69E+09 | 64189526 | 9.69E+09 | 99.98% | 9.32E+09 | 96.14% | 8.81E+09 | 90.94% |
| 2 yrs-3-STRI | 1.25E+10 | 82633290 | 1.25E+10 | 99.98% | 1.2E+10 | 96.01% | 1.13E+10 | 90.73% |
| 2 yrs-4-CERE | 1.25E+10 | 82548568 | 1.25E+10 | 99.98% | 1.21E+10 | 96.75% | 1.15E+10 | 91.88% |
| 2 yrs-4-CTX | 1.16E+10 | 76980140 | 1.16E+10 | 99.98% | 1.12E+10 | 96.73% | 1.07E+10 | 91.89% |
| 2 yrs-4-HIPP | 1.15E+10 | 76222172 | 1.15E+10 | 99.98% | 1.1E+10 | 95.96% | 1.04E+10 | 90.63% |
| 2 yrs-4-STRI | 1.15E+10 | 76431460 | 1.15E+10 | 99.98% | 1.11E+10 | 95.95% | 1.05E+10 | 90.59% |
| 8 yrs-1-CERE | 8.31E+09 | 55042590 | 8.31E+09 | 99.97% | 7.85E+09 | 94.48% | 7.3E+09 | 87.89% |
| 8 yrs-1-CTX | 6.52E+09 | 43176104 | 6.52E+09 | 99.97% | 6.18E+09 | 94.73% | 5.76E+09 | 88.37% |
| 8 yrs-1-HIPP | 9.32E+09 | 61683212 | 9.31E+09 | 99.97% | 8.83E+09 | 94.77% | 8.25E+09 | 88.53% |
| 8 yrs-1-STRI | 4.59E+09 | 30363680 | 4.58E+09 | 99.97% | 4.34E+09 | 94.59% | 4.04E+09 | 88.12% |
| 8 yrs-2-CERE | 9.51E+09 | 62971684 | 9.51E+09 | 99.97% | 9E+09 | 94.64% | 8.39E+09 | 88.23% |
| 8 yrs-2-CTX | 1.08E+10 | 71674060 | 1.08E+10 | 99.97% | 1.03E+10 | 94.97% | 9.6E+09 | 88.69% |
| 8 yrs-2-HIPP | 9.55E+09 | 63251410 | 9.55E+09 | 99.97% | 9.1E+09 | 95.23% | 8.52E+09 | 89.19% |
| 8 yrs-2-STRI | 9.48E+09 | 62784072 | 9.48E+09 | 99.97% | 9.01E+09 | 95.03% | 8.43E+09 | 88.88% |
| 8 yrs-3-CERE | 7.21E+09 | 47732494 | 7.21E+09 | 99.97% | 6.93E+09 | 96.15% | 6.55E+09 | 90.87% |
| 8 yrs-3-CTX | 7.47E+09 | 49447690 | 7.47E+09 | 99.98% | 7.15E+09 | 95.73% | 6.73E+09 | 90.10% |
| 8 yrs-3-HIPP | 9.23E+09 | 61092814 | 9.23E+09 | 99.97% | 8.83E+09 | 95.76% | 8.32E+09 | 90.14% |
| 8 yrs-3-STRI | 1.42E+10 | 94101834 | 1.42E+10 | 99.98% | 1.35E+10 | 95.35% | 1.27E+10 | 89.57% |
| 8 yrs-4-CERE | 1.27E+10 | 84348292 | 1.27E+10 | 99.98% | 1.22E+10 | 95.91% | 1.15E+10 | 90.26% |
| 8 yrs-4-CTX | 1.55E+10 | 1.03E+08 | 1.55E+10 | 99.98% | 1.47E+10 | 94.89% | 1.38E+10 | 88.82% |
| 8 yrs-4-HIPP | 1.17E+10 | 77785672 | 1.17E+10 | 99.98% | 1.13E+10 | 96.21% | 1.07E+10 | 90.91% |
| 8 yrs-4-STRI | 7.78E+09 | 51537512 | 7.78E+09 | 99.98% | 7.42E+09 | 95.32% | 6.95E+09 | 89.31% |
| 17 yrs-1-CERE | 1.08E+10 | 71489994 | 1.08E+10 | 99.97% | 1.02E+10 | 94.92% | 9.59E+09 | 88.88% |
| 17 yrs-1-CTX | 1.23E+10 | 81174018 | 1.23E+10 | 99.97% | 1.17E+10 | 95.62% | 1.11E+10 | 90.15% |
| 17 yrs-1-HIPP | 6.68E+09 | 44227020 | 6.68E+09 | 99.97% | 6.35E+09 | 95.08% | 5.95E+09 | 89.17% |
| 17 yrs-1-STRI | 1.12E+10 | 74128924 | 1.12E+10 | 99.97% | 1.06E+10 | 94.92% | 9.96E+09 | 89.02% |
| 17 yrs-2-CERE | 1.21E+10 | 80326316 | 1.21E+10 | 99.97% | 1.14E+10 | 94.37% | 1.07E+10 | 87.83% |
| 17 yrs-2-CTX | 1.07E+10 | 71039754 | 1.07E+10 | 99.97% | 1.01E+10 | 94.35% | 9.41E+09 | 87.69% |
| 17 yrs-2-HIPP | 8.78E+09 | 58115662 | 8.78E+09 | 99.97% | 8.3E+09 | 94.54% | 7.73E+09 | 88.11% |
| 17 yrs-2-STRI | 1.4E+10 | 92893124 | 1.4E+10 | 99.97% | 1.34E+10 | 95.44% | 1.26E+10 | 89.68% |
| 17 yrs-3-CERE | 9.37E+09 | 62010470 | 9.36E+09 | 99.97% | 8.92E+09 | 95.23% | 8.37E+09 | 89.37% |
| 17 yrs-3-CTX | 1.08E+10 | 71679408 | 1.08E+10 | 99.98% | 1.03E+10 | 95.21% | 9.66E+09 | 89.27% |
| 17 yrs-3-HIPP | 8.26E+09 | 54696090 | 8.26E+09 | 99.98% | 7.83E+09 | 94.80% | 7.32E+09 | 88.68% |
| 17 yrs-3-STRI | 7.9E+09 | 52302506 | 7.9E+09 | 99.98% | 7.42E+09 | 94.01% | 6.91E+09 | 87.43% |
| 17 yrs-4-CERE | 9.04E+09 | 59875518 | 9.04E+09 | 99.98% | 8.74E+09 | 96.62% | 8.29E+09 | 91.70% |
| 17 yrs-4-CTX | 7.52E+09 | 49760942 | 7.51E+09 | 99.98% | 7.22E+09 | 96.13% | 6.83E+09 | 90.90% |
| 17 yrs-4-HIPP | 5.99E+09 | 39690288 | 5.99E+09 | 99.98% | 5.73E+09 | 95.67% | 5.4E+09 | 90.12% |
| 17 yrs-4-STRI | 8.27E+09 | 54743876 | 8.27E+09 | 99.98% | 7.85E+09 | 94.98% | 7.36E+09 | 89.07% |

**Supplementary Table 3. The summary of aging-associated genes with DhMRs.**

| Tissue | Total genes | DhMR genes | age-associated genes | age-associated DhMR genes | Pearson's Chi-squared Test |  |
| --- | --- | --- | --- | --- | --- | --- |
| Cerebellum | 26,444 | 2,540 | 734 | 89 | 2.675E-02 | |
| Cortex | 26,444 | 3,162 | 734 | 119 | 5.972E-04 |  |
| Hippocampus | 26,444 | 5,979 | 734 | 245 | 1.015E-11 |  |
| Striatum | 26,444 | 9,652 | 734 | 310 | 1.680E-03 |  |
| Total | 26,444 | 13,600 | 734 | 435 | 3.290E-05 |  |

**Supplementary Table 4. Tissue-specific DhMRs between cerebellum and other regions.**

| DhMRs | Cere-specific | Brain-specific |
| --- | --- | --- |
| 2yrs | 29743 | 10394 |
| 8yrs | 20151 | 14027 |
| 17yrs | 15749 | 9367 |

**Supplementary Table 5. Dynamic and stable DhMRs in each brain region.**

| DhMRs | 2yrs-specific | 2yrs+8yrs-stable | 8yrs-specific | 8yrs+17yrs-stable | 17yrs-specific |
| --- | --- | --- | --- | --- | --- |
| Cerebellum | 901 | 364 | 298 | 1082 | 2836 |
| Hippocampus | 1301 | 274 | 5674 | 622 | 5471 |
| Striatum | 4415 | 9910 | 3988 | 446 | 24619 |
| Cortex | 696 | 70 | 1507 | 1122 | 2885 |

**Supplementary Table 6. GO term of DhMRs of striatum in 17 years group.**

| **GO ID** | **Description** | **Genes** | **P-value** |
| --- | --- | --- | --- |
| GO:0007612 | learning | FGF13;AAAS;NRXN3;ATP8A1;CNTNAP2;NRXN1;AMPH;DRD3;GRM5;NLGN4Y; | 0.000185 |
| GO:0008542 | visual learning | KIT;NDRG4;BRAF;PDE8B;DRD2;DRD3;DCDC2;HTT; | 0.001146 |
| GO:0042297 | vocal learning | FOXP2;CNTNAP2;NRXN1; | 0.004209 |
| GO:0007613 | memory | FGF13;GIP;ITGA5;PLCB1;PDE4D;CHRNA7;ITGA8;IL1RN; | 0.004587 |
| GO:0050890 | cognition | SOBP;CHRNA7;GRM5;TUSC3;DOPEY2; | 0.023413 |
| GO:0007616 | long-term memory | DRD2;GRIA1;CALB1;RELN; | 0.031735 |
| GO:0007614 | short-term memory | GRM7;CALB1; | 0.066535 |
| GO:0007626 | locomotory behavior | SOBP;ATP7A;MCOLN3;CACNA1B;NPAS3;DRD3;GRM5;NEGR1;HTR2C;GRM6;ABAT;DRD2;ALS2;CALB1;HTT;CELSR1;CHRNA3; | 3.61E-07 |
| GO:0007628 | adult walking behavior | CACNA1A;KCNMA1;DAB1;KLHL1;DRD2;GBX1;SCN8A;CACNB4;PCDH15; | 6.38E-05 |
| GO:0008344 | adult locomotory behavior | OPRD1;GIP;FGF12;TSHR;PBX3;FGF14; | 0.023685 |
| GO:0035641 | locomotory exploration behavior | PRKCE;LSAMP; | 0.077824 |
