# Supplementary figures and images for: Brain Region- and Age-Dependent 5-Hydroxymethylcytosine Activity in the Non-Human Primate
Source: Front Aging Neurosci. 2022 Jul 13;14:934224. doi: 10.3389/fnagi.2022.934224 (PMC9326314; doi:10.3389/fnagi.2022.934224)

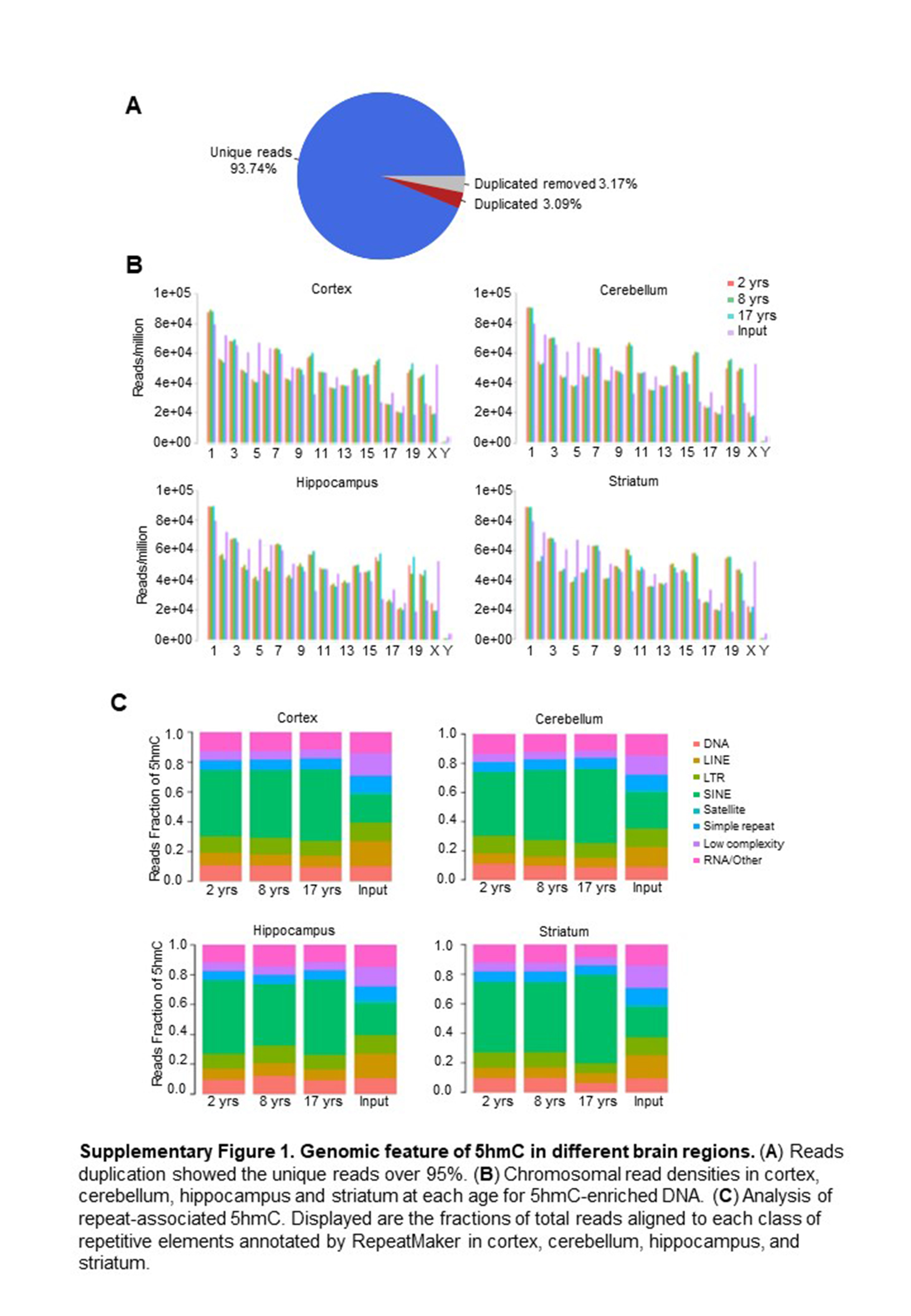

Supplement: Supplementary file 2 [file Image_1.JPEG]

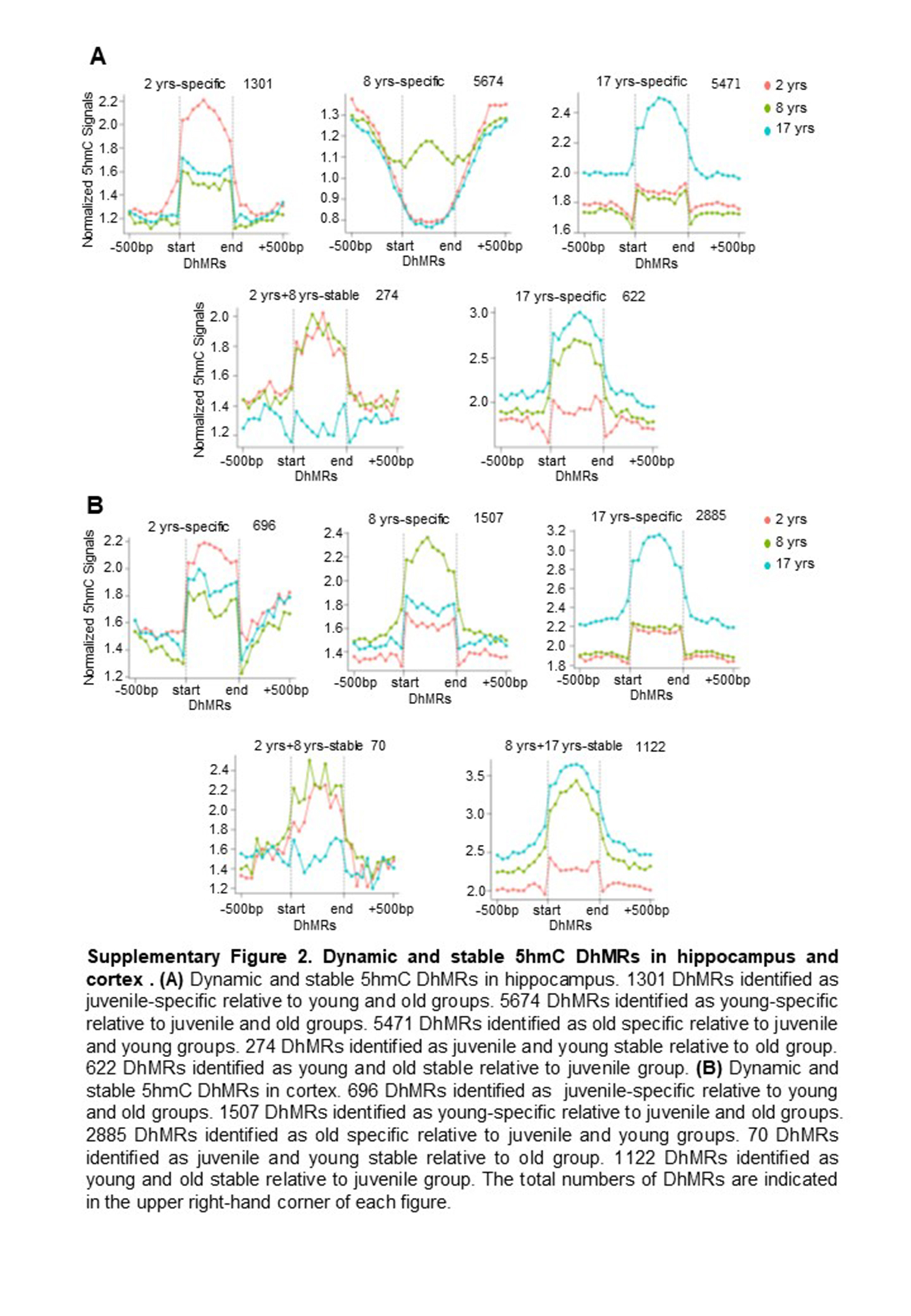

Supplement: Supplementary file 3 [file Image_2.JPEG]

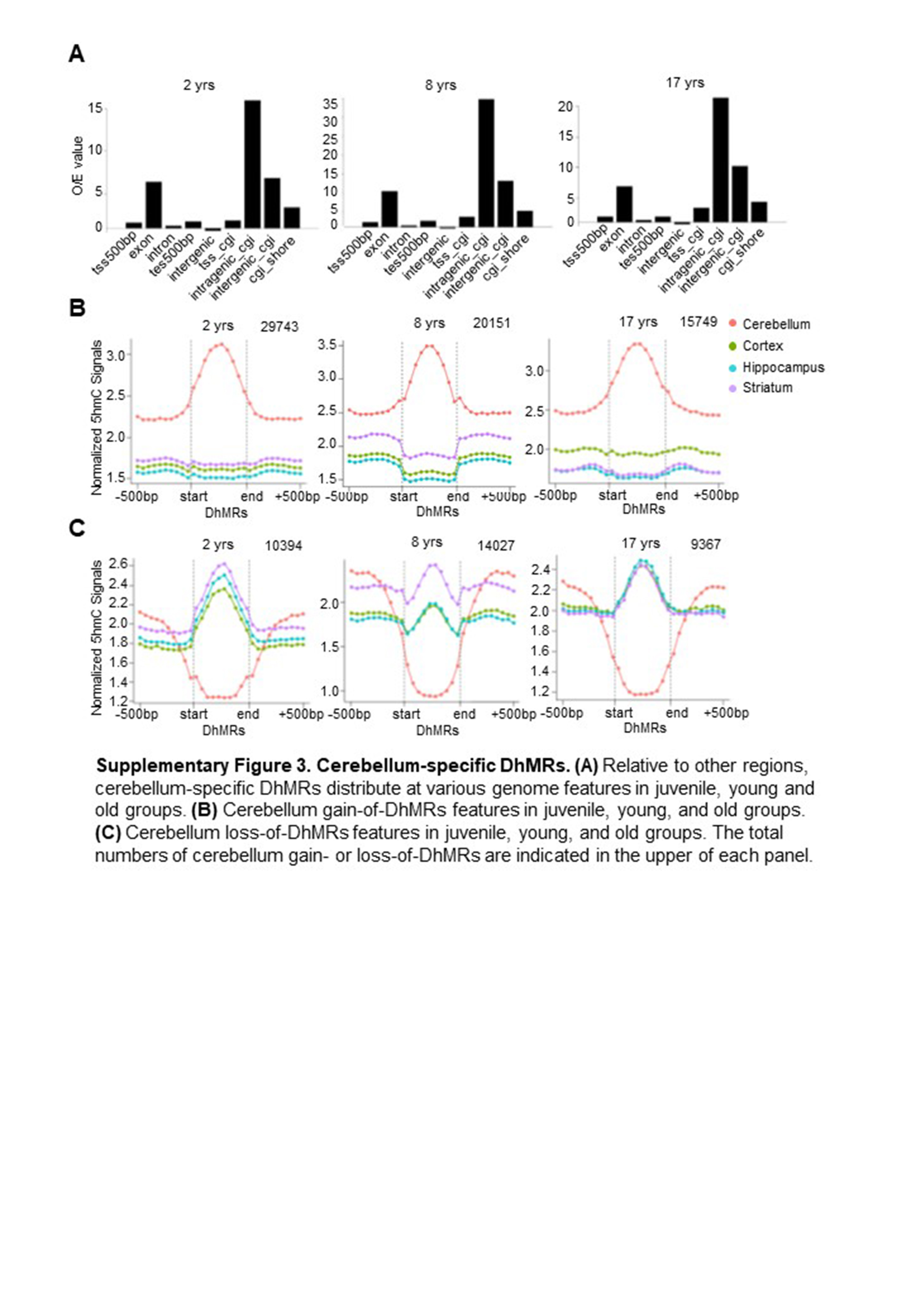

Supplement: Supplementary file 4 [file Image_3.JPEG]

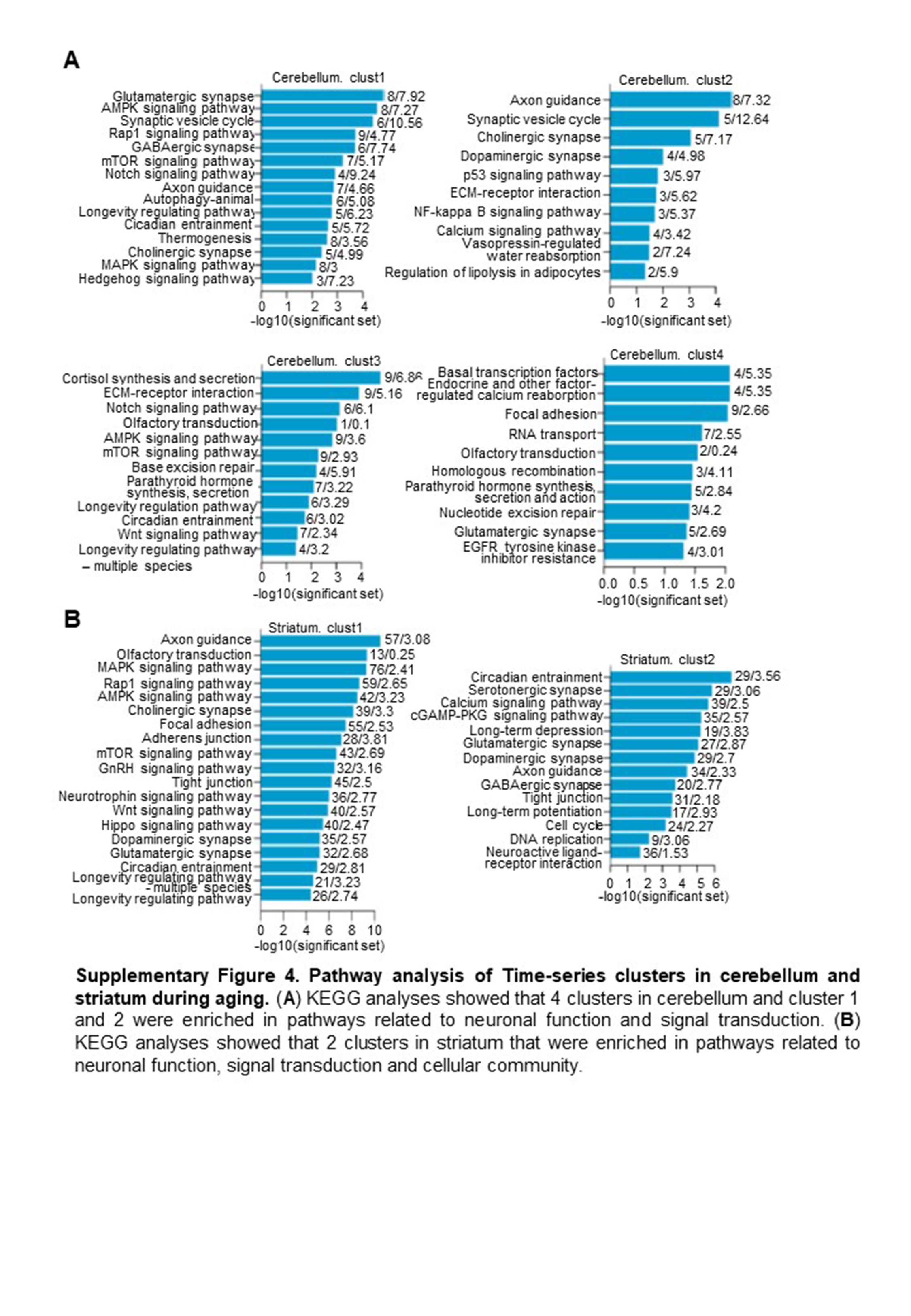

Supplement: Supplementary file 5 [file Image_4.JPEG]

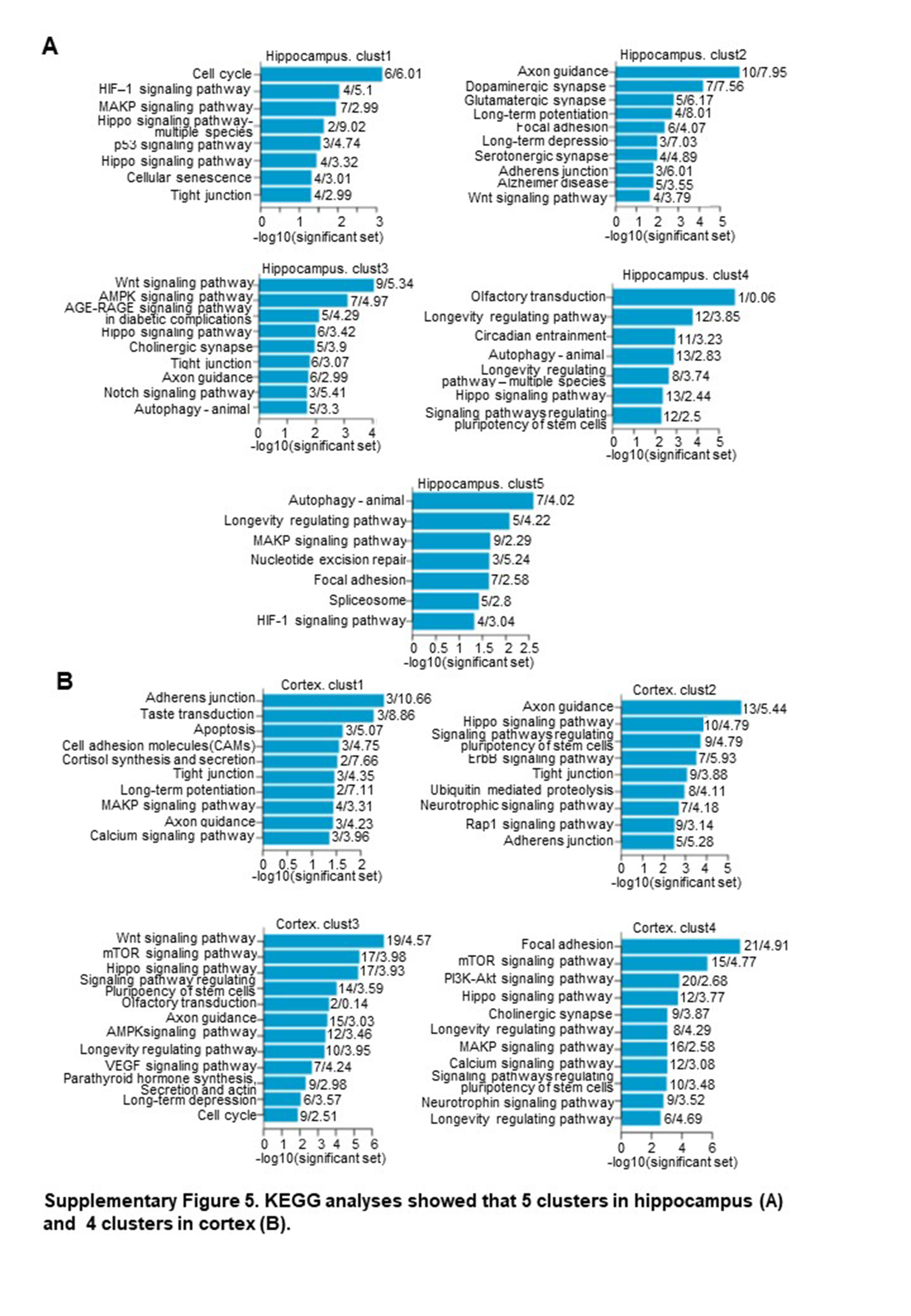

Supplement: Supplementary file 6 [file Image_5.JPEG]
